# Aberrant Activation of Heat Shock Protein 60/65 Reactive T Cells in Patients with Behcet's Disease

**DOI:** 10.1155/2012/105205

**Published:** 2012-10-02

**Authors:** Jun Shimizu, Tomoko Izumi, Noboru Suzuki

**Affiliations:** ^1^Department of Immunology and Medicine, St. Marianna University School of Medicine, 2-16-1, Sugao, Miyamae-ku, Kawasaki 216-8511, Japan; ^2^Department of Medicine, Self-Defense Forces Central Hospital, Tokyo 154-8532, Japan

## Abstract

Behcet's disease (BD) is a multisystemic inflammatory disease and is characterized by recurrent attacks on eyes, brain, skin, and gut. There is evidence that skewed T-cell responses contributed to its pathophysiology in patients with BD. We found that heat shock proteins (HSPs) reactive T cells were prevalent in patients with BD. Here, we summarize current findings on HSP reactive T cells and their contribution to the pathogenesis in patients with BD.

## 1. Introduction 

Behcet's disease (BD) is a systemic inflammatory disease, characterized by recurrent signs and symptoms of oral aphthosis, genital ulcers, skin lesions, and uveitis. It is well known that BD is prevalent along the Silk Route, but BD patients are occasionally found in other regions of the world. 

The etiology of BD is largely unknown and skewed T-cell responses are associated with development and maintenance of BD [1]. Excessive cytokine productions by T helper type 1 (Th1) cells were reported using immunohistochemistry [2, 3] and intracellular cytokine staining [4, 5]. Th1 dominance was observed in BD uveitis [6] and stomatitis as well [7]. We reported excessive Th1 cell infiltration in BD skin and intestinal lesions [8–10]. 

Immune responses against microbes and microbial antigens were thought to play an important role in the pathogenesis of BD. Regional differences of the disease distribution [11] suggested association of disease development with locally prevalent microbes. Oral health was often impaired in patients with BD and was correlated well with BD disease severity [12]. *Streptococcus sanguinis* is a commensal oral bacterium and often forms dental plaque. *S. sanguinis* was found frequently in oral flora in patients with BD and the strain showed uncommon serotype (KTH1) compared with the standard ATCC strains [13]. T cells and peripheral blood mononuclear cells (PBMCs) from patients with BD responded to KTH1 antigens and produced interferon *γ* (IFN*γ*) and interleukin (IL)-12 [14]. Skin tests of streptococcal antigens caused various systemic reactions, such as fever, ocular attack, and genital and oral ulcer in patients with BD [15]. Accumulation of indirect evidence suggested participation of bacteria (or associated antigens) in the pathogenesis of BD. 

Pathergy reaction is a cutaneous phenomenon where a minor injury, such as a needle prick, causes major skin lesions, such as ulcerations, panniculitis, and pyoderma, and positive pathergy reaction is included in the diagnostic criteria for BD proposed by the International Study Group [16]. Massive neutrophil infiltration and subsequent T-cell infiltration were frequently observed pathologically in the lesion caused by the reaction, even without any exogenous microbes [2]. The underlying mechanisms of the reaction remain largely unknown. On the other hand, it was suggested that skin florae and some skin self-antigens played a role because bacterial sterilization of skin reduced the reaction [17].

Heat shock proteins (HSPs) function as an intracellular chaperonin for other proteins, and significant sequence homology is found between mammalian HSP and microbial HSP (Table 1). For example, mycobacterial and streptococcal HSP65 have more than 90% homology, and mycobacterial HSP65 and human HSP60 have 42% homology [18]. HSP60/65 were thought to be a major cause of the autoimmunity in patients with BD because of the molecular mimicry between human and microbial HSP. In this paper, we summarize current understanding of T-cell responses against HSP in patients with BD.

## 2. HSP Expressions in BD Lesions

Lehner et al. found that monoclonal antibodies against HSP65 reacted with six *S. sanguinis* strains and *Streptococcus pyogenes* [19]. They revealed that both IgG and IgA antibodies against HSP65 and *S. sanguinis* were significantly increased in BD patients compared to normal controls. They showed molecular mimicry between HSP and streptococcal antigens and suggested HSP-antigen-specific autoimmunity in the pathogenesis of BD. 

After the initial report, researchers tried to identify the expression of HSP and to analyze immune cell functions on biopsy specimens in patients with BD. Several researchers observed massive expressions of HSP60 in BD skin [23] and oral ulcer lesions [24, 25]. HSP60 was expressed more diffusely [25] and intensely [23, 25] in BD lesions than those in other types of inflammation, such as oral lichen planus and recurrent aphthous stomatitis. Not only infiltrating cells but also vascular endothelial and epithelial cells expressed HSP60 in the BD skin lesions [25].

We reported excessive Th1 cell function and aberrant HSP expression in patients with BD [8–10]. HSP60 mRNA expression was found in PBMC and in intestinal tissues of BD but not in those of normal controls [8]. We found that infiltrating mononuclear cells, including CD4+ T cells, CD8+ T cells, and CD68+ macrophages, expressed HSP60, IFN*γ*, and IL-12 in the intestinal lesions in patients with BD [8, 9]. C-C type chemokine receptor (CCR)5 and macrophage inflammatory protein (MIP)1*β*, a Th1-related chemokine receptor and its ligand, were detected in the intestinal lesions in patients with BD, and we suggested that CCR5/MIP1*β* interaction played a role in the migration of activated Th1 cells [8]. It is possible that HSP60 acted as a crucial signal to trigger excessive Th1 cell accumulation and subsequent Th1-cell-mediated inflammatory responses in the intestinal lesions in patients with BD where activated T cells and macrophages promoted the destructive processes.

## 3. T-Cell Responses against HSP Peptides in Patients with BD

**Figure 1:**
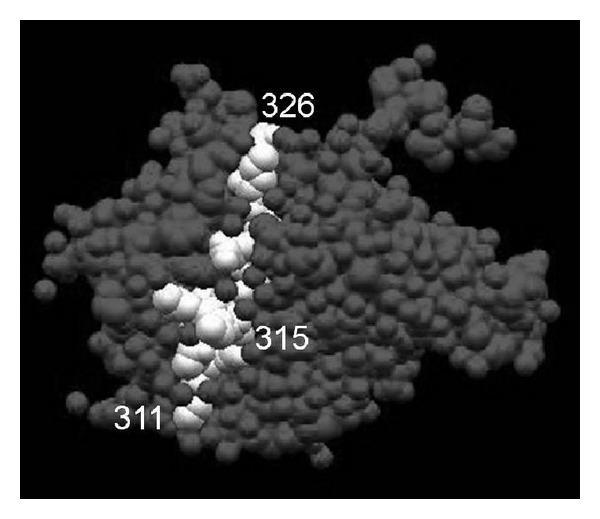
Crystal structure of HSP. GroEL fragment apical domain, a bacterial homologue of human HSP 60, comprising residues 191–345 derived from *Escherichia coli* in the Protein Data Bank Japan (PDBj) is shown. Residues 311–326 which corresponded to human HSP60 331–356 are colored with white.

Lehner et al. analyzed the frequency of short-term cell lines stimulated with four selected peptides (111–125, 154–172, 219–233, 311–326) (Figure 1, Table 2) derived from *Mycobacterium tuberculosis* HSP65 in the ocular, arthritic, and mucocutaneous types of BD. T cells from ocular-type BD patients responded excessively to the stimulation with the four HSP peptides, especially peptides 111–125 and 311–326 [20]. 

Excessive T- and B-cell responses to the four peptides and human counterparts were observed in patients with BD who lived in Europe, Far Eastern Asia, and the Middle East [10, 20–22]. 

In our study, CD4+ T cells, but not CD8+ T cells, yielded proliferative responses to the peptide 336–351 derived from human HSP60 in Japanese BD patients [26]. We evaluated T-cell receptor V*β* gene usage of T cells which responded to the peptide 336–351 in the patients [22]. To this end, we first conducted amplification by PCR of TCR V*β* gene of T cells which had been stimulated with the peptide *in vitro* and then visualized several bands which represented each TCR clonotype by PCR-single-strand conformation polymorphism (SSCP) based technique. T-cell receptor V*β* gene oligoclonality was found in T cells which had been freshly isolated from peripheral blood and the exactly same PCR products were remarkably increased after stimulation with the peptide 336–351 in patients with BD. These data suggested that HSP-specific T cells in patients with BD showed antigen-driven expansion by HSP stimulation and that the HSP peptide reactive T cells increased in BD peripheral blood. A longitudinal study of the TCR clonotypes in 6 BD patients showed that the oligoclonal expansion of particular T-cell clonotypes correlated with the severity of uveitis [22].

## 4. **γ** **δ** T-Cell Activation and HSP60/65

HSP60/65 presented by antigen presenting cells (APCs) stimulated not only *αβ* T cells but also *γδ* T cells which played important roles in the oral mucosal immunity as the first defense against microorganisms. It was thought that V*γ*9*δ*2+ T cells, a major subset of *γδ* T cells of peripheral blood lymphocytes (PBL), recognized the antigens produced by bacteria [26]. V*δ*1+ *γδ* T cells responded to the stress inducible major histocompatibility complex class I related chain A (MICA) mainly expressed on damaged intestinal epithelial cells [28].

V*δ*2+ *γδ* T cells increased in peripheral blood, while V*δ*1+ *γδ* T cells increased in bronchoalveolar lavage fluid and cerebrospinal fluid in patients with BD [29]. Infiltrating cells expressed HSP, and *γδ* T-cell numbers were increased in oral ulcer in the patients [23, 25]. 

We found that CD45RA+ V*γ*9*δ*2+ *γδ* T cells increased in BD PBL irrespective of disease activity. V*γ*9*δ*2+ *γδ* T cells in the active phase of BD expressed IL-2 receptor *β* chain and HLA-DR, suggesting that the cells were activated *in vivo* [30]. The CD45RA+ *γδ* T cells produced tumor necrosis factor (TNF)*α* and contained perforin granules. Moreover, V*γ*9*δ*1+ *γδ* T cells preferentially responded to *S. sanguinis*-derived KTH1 antigen without HLA restriction [31]. It is possible that *γδ* T cells respond to HSP both in peripheral blood and in affected lesions and enhance the inflammation in patients with BD.

## 5. Th17 Cells and HSP

**Figure 2:**
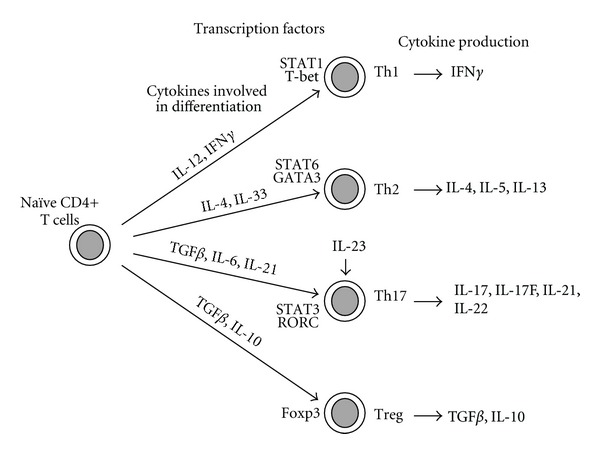
Current view of CD4+ T-cell subsets in humans [27]. Naïve CD4+ T-cells differentiate into several T-cell subsets in the presence of appropriate cytokines. In response to the cytokines, the corresponding signaling molecules and transcription factors are expressed to regulate lineage commitments. Th17 cells require IL-23 for their expansion.

Recently, the classical Th1/Th2 paradigm was challenged by the discovery of various subsets of T helper cells [27] (Figure 2). Th17 cells produce a number of proinflammatory cytokines, including IL-17, IL-17F, IL-21, and IL-22. IL-6, IL-21, and transforming growth factor (TGF)*β* were reported to play a role in the differentiation of Th17 cells which proliferated in the presence of IL-23. Regulatory T (Treg) cells control T-cell immune responses and also need TGF*β* for their differentiation [27] (Figure 2). 

**Figure 3:**
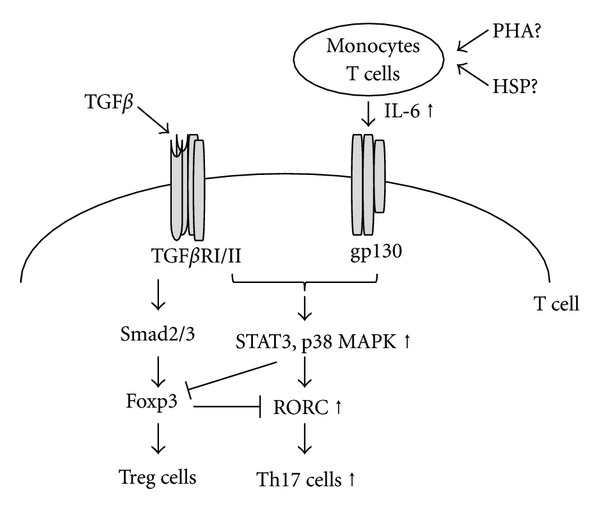
A schematic representation of skewed Th17/Treg cell differentiation in patients with BD, a hypothesis. TGF*β* activates Smad pathway via TGF*β* receptor I/II complex, and activated Smad protein leads to forkhead box P3 (Foxp3) expression which is a master gene of Treg cells [27]. TGF*β* also activates p38 mitogen-activated protein kinase (MAPK) which regulates Th17 cell differentiation [32, 33]. In the presence of TGF*β*, IL-6/STAT3 signaling pathway plays a critical role in the reduction of Foxp3 expression and in the induction of retinoic acid receptor-related orphan receptor C (RORC) expression which is a master gene of Th17 cells [34]. In patients with BD, monocytes and T cells overproduce IL-6 in the presence of HSP [41]. Overexpression of RORC [42, 43], underexpression of Foxp3 [44, 45], and higher frequencies of Th17 cells [42–44, 46] are reported in patients with BD. PHA: phytohemagglutinin, TGF*β*RI/II: TGF*β* receptor types I and II, STAT3: signal transducer and activator of transcription 3.

TGF*β* activates Smad pathway via TGF*β* receptor I/II complex and activated Smad protein leads to forkhead box P3 (Foxp3) expression which is a master gene of Treg cells [32] (Figure 3). TGF*β* also activates p38 mitogen-activated protein kinase (MAPK) which regulates Th17 cell differentiation [32, 33]. In the presence of TGF*β*, IL-6/STAT3 signaling pathway plays a critical role in the reduction of Foxp3 expression and in the induction of retinoic acid receptor-related orphan receptor C (RORC) expression which is a master gene of Th17 cells [34] (Figure 3). Also it is reported that STAT3 and MAPK are involved in the immune tolerance induction and Treg cell differentiation [35, 36]. Signaling molecules such as STAT3 and MAPK may transduce positive and negative signals depending upon the surrounding microenvironment. To address these issues, further studies are needed. In addition, specific antigen such as HSP directly and indirectly regulated the balance between Th17 and Treg cells [37–40].

In patients with BD, monocytes and T cells overproduced IL-6 in the presence of HSP [41]. Overexpression of RORC mRNA [42, 43], underexpression of Foxp3 [44, 45], and high frequencies of Th17 cells [42–44, 46] were reported in patients with BD. We recently reported that TGF*β*/Smad signaling pathway of T cells was overactivated in patients with BD [47]. We also reported the possibility that CD4+ T cells in patients with BD showed higher sensitivity to IL-23 and produced more IFN*γ* and IL-17, as compared with normal controls [43]. Recent genome-wide association studies identified IL-12 receptor *β*2 (IL-12R*β*2)/IL-23 receptor and IL-10 genes as BD susceptibility genes [48, 49]. Based on the above findings, we proposed that HSP induced imbalance of Th17 cells to Treg cells in patients with BD (Figure 3). Further study on HSP and Th17 cell differentiation is necessary to understand the pathogenesis of BD.

## 6. Toll-Like Receptors (TLRs) and HSP60/65

TLRs play a key role in recognition of microbes in the innate immune system. Activation of dendritic cells by TLR ligands is a crucial event in the initiation of both innate and adaptive immune responses. Several classes of TLR ligands are identified which interact with distinct members of the TLR family. TLR2/CD14 complex and TLR4/CD14 complex were suggested to be important for APC to recognize HSP60 [50], and TLR2/6 heterodimer was reported to promote IL-6 production of mononuclear cells [51]. We need to study the expression of the heterodimer because IL-6 concentrations were elevated in patients with BD [41].

We found that TLR2 and TLR4 mRNA were expressed on ileocaecal ulcer lesions of BD, but less on unaffected sites of BD and on Crohn's disease lesions. We found that IL-12 producing TLR2+ macrophages are located neighboring to CD3+ T cells. HSP60 was expressed on the same region of the intestinal lesions [9]. We suggested that TLR/HSP60 interactions induced destructive Th1-type responses at the intestinal lesion in patients with BD. 

## 7. Conclusions 

**Figure 4:**
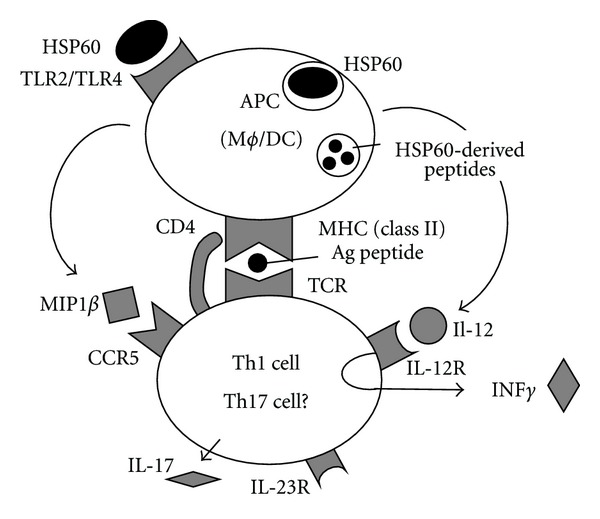
HSP60 regulates Th1 cell differentiation through multiple pathways, a hypothesis. The interactions between TCR- and HSP-derived peptides on MHC [22] and between IL-12 and its receptor [8–10] may induce aberrant T-cell differentiation in patients with BD (Figure 4). The interaction between MIP1*β* and C-C-type chemokine receptor (CCR)5 was suggested to induce migration of the pathogenic T cells [8]. TLR2/6 heterodimer is involved in IL-6 production [51] and thus we need to study the expression of the heterodimer in patients with BD.

We reviewed here HSPs reactive T-cells and their contribution to the pathogenesis of BD. It is possible that HSPs regulate T-cell differentiation through several interactions. The interactions between TCR- and HSP-derived peptides on MHC [22] and between IL-12 and its receptor [8–10] may induce aberrant T-cell differentiation in patients with BD (Figure 4). The interaction between MIP1*β* and CCR5 was suggested to induce migration of the pathogenic T cells [8]. TLR2/6 heterodimer is involved in IL-6 production [51] and thus we need to study the expression of the heterodimer in patients with BD. It is important to clarify whether HSP bring about generation of pathogenic Th17 cells in patients with BD because Th1 and Th17 cells share a common structure (IL-12R*β*1) on their characteristic receptor complexes, namely, IL-12R and IL-23R [52].

## Figures and Tables

**Table 1:** Comparison of the amino acid sequences of human, Chinese hamster (C) HSP60, *Escherichia coli* (E), and *Mycobacterium leprae* (M) HSP65 [18].

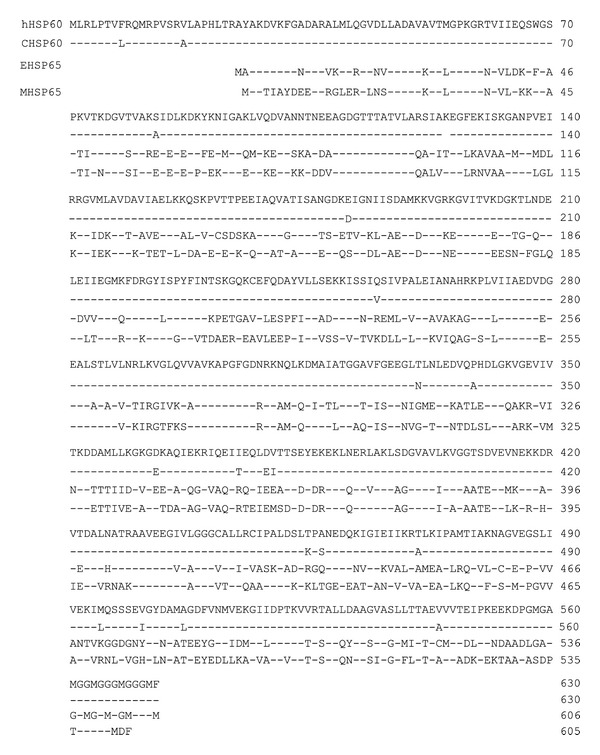

Residues identical to the human HSP are indicated by dash. Significant sequence homology is found between mammalian and microbial HSP. *E. coli*-derived GloEL fragment and human HSP60 have 45.2% homology.

**Table 2:** Conserved peptide sequences of mycobacterial HSP65 and human HSP60 [20].

Mycobacterial HSP65	(111–125)	N	P	L	G	L	K	R	G	I	E	K	A	V	E	K				
Human HSP60	(136–150)	N	P	V	E	I	R	R	G	V	M	L	A	V	D	A				
Mycobacterial HSP65	(154–172)	Q	S	I	G	D	L	I	A	F	A	M	D	K	V	G	N	E	G	V
Human HSP60	(179–197)	K	E	I	G	N	I	I	S	D	A	M	K	K	V	G	R	K	G	V
Mycobacterial HSP65	(219–233)	L	L	V	S	S	K	V	S	T	V	K	D	L	L	P				
Human HSP60	(244–258)	L	L	S	E	K	K	I	S	S	I	Q	S	I	V	P				
Mycobacterial HSP65	(311–326)	D	L	S	L	L	G	K	A	R	K	V	V	V	T	K	D			
Human HSP60	(336–351)	Q	P	H	D	L	G	K	V	G	E	V	I	V	T	K	D			

The four peptide sequences were well conserved between human HSP60 and mycobacterial HSP65. Peptide 336–351 derived from human HSP60 effectively stimulated the pathogenic T cells in patients with BD [10, 20–22].
